# Crimean-Congo Hemorrhagic Fever Virus in Ticks from Migratory Birds, Morocco^1^

**DOI:** 10.3201/eid1902.121193

**Published:** 2013-02

**Authors:** Ana M. Palomar, Aránzazu Portillo, Paula Santibáñez, David Mazuelas, Juan Arizaga, Ariñe Crespo, Óscar Gutiérrez, Juan Francisco Cuadrado, José A. Oteo

**Affiliations:** Author affiliations: Hospital San Pedro–CIBIR, Center of Rickettsioses and Arthropod-Borne Diseases, Logroño, Spain. (A.M. Palomar, A. Portillo, P. Santibáñez, J.A. Oteo);; Environment Resources Inc., Logroño (D. Mazuelas);; Aranzadi Society of Sciences, San Sebastián, Spain (J. Arizaga, A. Crespo, Ó. Gutiérrez, J.F. Cuadrado)

**Keywords:** Crimean-Congo hemorrhagic fever virus, ticks, migratory birds, Morocco, Spain, Europe, vector-borne infections, viruses, birds

## Abstract

Crimean-Congo hemorrhagic fever virus was detected in ticks removed from migratory birds in Morocco. This finding demonstrates the circulation of this virus in northwestern Africa and supports the hypothesis that the virus can be introduced into Europe by infected ticks transported from Africa by migratory birds.

Crimean-Congo hemorrhagic fever virus (CCHFV), the causative agent of Crimean-Congo hemorrhagic fever (CCHV), is an arthropod-borne virus (arbovirus) with clinical relevance worldwide (1). CCHF causes sudden onset of signs and symptoms including headache, high fever, back pain, joint pain, stomach pain, and vomiting, which can progress to severe bruising, severe nosebleeds, and uncontrolled bleeding (www.cdc.gov/ncidod/dvrd/spb/mnpages/dispages/cchf.htm).

CCHFV, belonging to the genus *Nairovirus*, circulates in an enzootic tick-vertebrate-tick cycle in which ticks can act as vectors and reservoirs. CCHFV has been found in ticks of >30 species; *Hyalomma marginatum* ticks are considered the most common vectors. Birds are the main hosts for the immature stages of this tick species (2). Viremia does not develop in most passerine birds (3,4), which are not able to pass the virus to ticks. However, migratory species could carry infected ticks over long distances and thereby disseminate the virus (2).

Since the first descriptions of human infections with this virus in 1944–1955 in Crimea, outbreaks of CCHF have been reported in Africa, Asia, and eastern Europe (1). Only imported cases have been reported in western Europe, although the causal agent has been amplified in *H. lusitanicum* ticks collected from deer in Spain (southwestern Europe) (5). This finding could be explained by the arrival of infected ticks transported by migratory birds coming from Africa (5). To confirm this hypothesis, we investigated the presence of CCHFV in ticks collected from migratory birds in northern Africa.

## The Study

**Figure 1:**
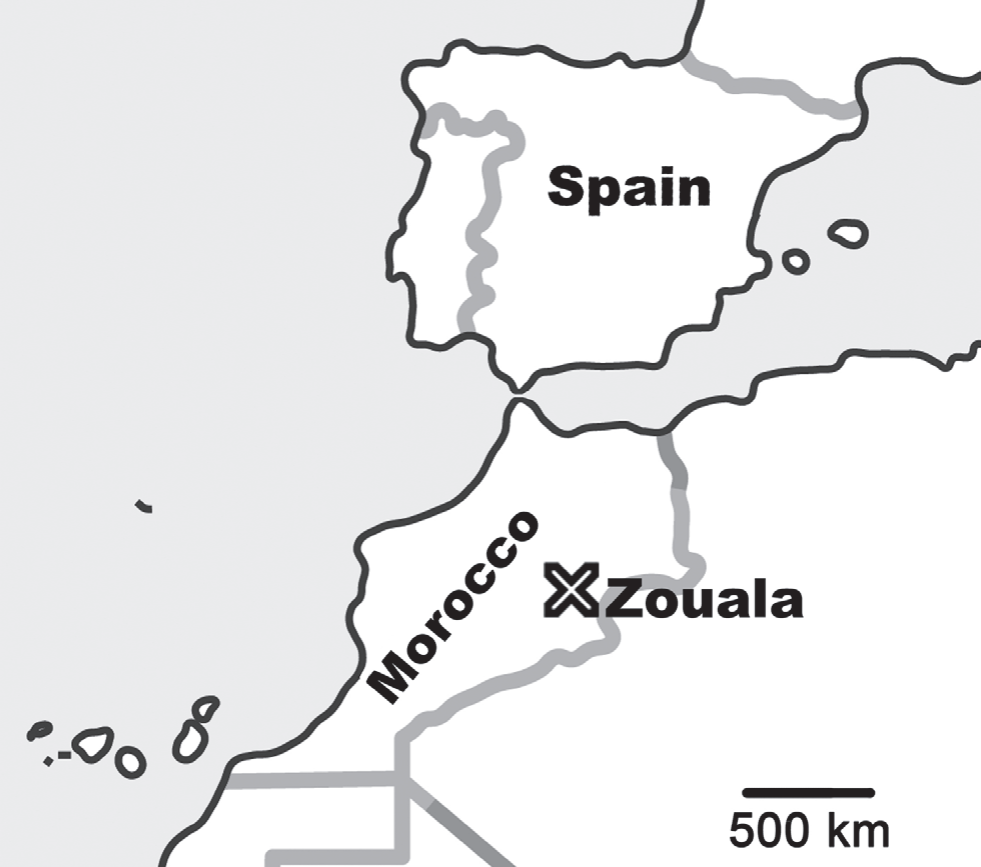
Location of Zouala, Morocco.

In April 2011, bird bandings were conducted in Zouala, Morocco (31°47′N, 4°14′W) (Figure 1). A total of 546 captured birds were checked for ticks, and parasites were found on 21 birds from 5 passerine bird species (*Phoenicurus phoenicurus*, *Erythropygia galactotes*, *Iduna opaca*, *Acrocephalus scirpaceus*, and *I. pallida*). All but *I. pallida* birds are passerine trans-Saharan migrant species, coming from central and southern Africa and able to reach the Iberian Peninsula.

A total of 52 ticks (19 larvae and 33 nymphs) were processed. Genomic DNA and total RNA from ticks were individually purified by using the AllPrep DNA/RNA Mini Kit (QIAGEN, Hilden, Germany), according to the manufacturer’s instructions. DNA extracts were used as templates for 16S rDNA PCR assays (6), and all specimens were classified as *H. marginatum* ticks. RNA was retrotranscribed by using the Omniscript RT kit (QIAGEN), according to the manufacturer’s instructions. cDNA extracts were distributed in 6 pools used as templates for 2 nested PCRs with Eecf and Gre primer pairs (7) (Table). Negative controls (with template DNA but without primers and with primers and containing water instead of template DNA) were included in all assays.

**Table:** Distribution in pools of *Hyalomma marginatum* ticks collected from birds in Zouala, Morocco, and PCR results for detection of Crimean-Congo hemorrhagic fever virus*

Pool	Bird species (no. specimens)	No. *H. marginatum* ticks collected, by stage			PCR results	
		Larvae	Nymphs		Eecf primers	Gre primers
A	*Erythropygia galactotes* (1)		2 FE		+	–
	*Phoenicurus phoenicurus* (1)		6 FE			
B	*E. galactotes* (2)	1 SE	7 FE		+	–
	*Iduna opaca* (1)	3 FE				
C	*I. opaca* (2)	6 FE	1 FE		+	–
	*P. phoenicurus* (1)		1 SE			
D	*Acrocephalus scirpaceus* (1)	4 SE	1 FE		+†	–
	*I. opaca* (1)		1 FE			
	*I. pallida* (1)		1 SE, 1 FE			
E	*A. scirpaceus* (1)	2 FE			–	–
	*E. galactotes* (1)		2 FE			
	*I. opaca* (4)	1 FE	2 SE, 1 FE			
	*P. phoenicurus* (1)		1 FE			
F	*E. galactotes* (1)		2 FE		–	–
	*I. opaca* (2)	2 SE	1 SE, 1 FE			
	*P. phoenicurus* (1)		1 SE, 1 FE			

*FE, fully engorged; +, positive; –, negative; SE, semi-engorged. †No sequence was obtained.

**Figure 2:**
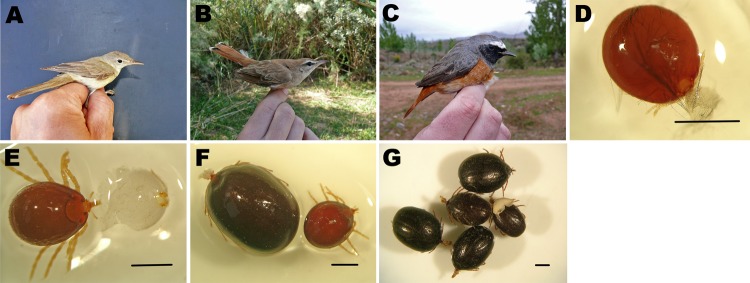
Bird species and tick specimens collected in Zouala, Morocco, April 2011. A) *Iduna opaca*, B) *Erythropygia galactotes,* and C) *Phoenicurus phoenicurus* birds. D–G) *Hyalomma marginatum* tick specimens removed from birds and preserved in alcohol: D) semi-engorged larva, E) semi-engorged nymph, F) semi-engorged and fully engorged nymphs, and G) fully engorged nymphs. Scale bars indicate 1 mm.

Nested PCR assays using Eecf primers were positive for 4/6 pools; all samples were negative by Gre PCR primers (7). Three of 4 amplicons (associated with *P. phoenicurus*, *E. galactotes*, and *I. opaca* bird species; Figure 2) could be sequenced, and nucleotide sequences were compared with those available in GenBank by using BLAST (www.ncbi.nlm.nih.gov/blast/Blast.cgi). All nucleotide sequences were identical and showed 100% identity with the Sudan AB1-2009 and Mauritania ArD39554 CCHFV strains (GenBank accession nos. HQ378179 and DQ211641) and 98.9% identity with the sequence amplified from ticks from Spain (5).

## Conclusions

The detection of CCHFV in ticks from migratory birds in Zouala demonstrates the circulation of this virus in Morocco. This country has optimal conditions for the establishment of CCHF, including populations of *H. marginatum* ticks and reservoirs of the virus, such as livestock. Furthermore, autochthonous cases of the disease have been reported in neighboring Mauritania (8).

Our finding of 3 positive tick pools demonstrates the potential dispersion of the virus through infected ticks transported by migratory birds. Several bird species, including *P. phoenicurus*, *E. galactotes*, *I. opaca*, and *A. scirpaceus*, migrate in the spring from southern or central Africa to northern Europe. The Iberian Peninsula may be a stopover or breeding site along those routes (9), which suggests that migratory birds may transport *H. marginatum* ticks from Africa to Europe (2).

Ticks of this species are found in Africa, southern Asia, and southern Europe (10,11); some specimens have been collected in northern European regions such as Germany or England (12,13). Birds are commonly parasitized by immature stages of *H. marginatum*, a 2-host tick that has the same host for larva and nymph stages. Thus, ticks may be attached to the bird for >2–3 weeks (11). An average ground speed of 50 km/h for migratory birds crossing Sahara has been reported (14); this speed would enable birds to cover the distance between Morocco and the Iberian Peninsula in less time than it would take ticks to develop from immature to adult.

In early May 2012, our team captured *A. scirpaceus* birds parasitized by *H. marginatum* ticks in northern Spain (42°48′N; 2°39′W); some ticks were fully engorged nymphs (A.M. Palomar, unpub. data). A high probability exists that the larvae were attached to birds in Africa and molted and engorged during migration, which supports the possibility of the arrival of migratory birds with CCHFV-infected ticks.

A study conducted in England of CCHFV in ticks collected from migratory birds found negative real-time PCR results for CCHFV, although the parasitization rate of birds with *H. marginatum* ticks was low (13). In addition, a group of experts has reported that migratory birds may not be sufficient to establish new foci of CCHFV infection in Europe and may not represent a high risk for its implantation because adult ticks are necessary and immature specimens cannot find optimal climate conditions to molt in spring (10). However, this report stated that Spain’s average spring temperatures are lower than those needed for birds to molt; in April 2011, the weather station located in northern Spain (La Rioja) (42°27′N, 2°19′W) recorded average temperatures >14°C for 20/30 days. In addition, *H. marginatum* tick populations are established on the Iberian Peninsula.

The finding of fragments of the small segment of CCHFV identical to fragments of the Mauritania and Sudan strains and closely related to the sequence previously amplified by our team (5), in pools of immature specimens of different engorged states (Figure 2), may explain 2 hypotheses. First, infected ticks from CCHF-endemic areas, such as Mauritania or Sudan, could have attached to birds and transported to Morocco over them. Second, these ticks could have been carried from Africa to the Iberian Peninsula, thus explaining the circulation of CCHFV in southwestern Europe.

Autochthonous cases of CCHF have not been reported in southwestern Europe, and CCHFV has not been detected in ticks from migratory birds in Europe (13). However, some authors support the limited role of migratory birds harboring CCHFV-infected ticks for the establishment of the virus (10,15). Nevertheless, the finding of CCHFV from migratory birds in Morocco, along with the previous detection of the virus in Spain (5), where *H. marginatum* tick populations and CCHFV reservoirs occur, call for further study of the distribution of CCHFV in southwestern Europe. Health care workers should be informed about the possibility of CCHF and the clinical picture in the potential disease-endemic areas, and groups potentially at risk for infection (e.g., health care workers, hunters, farmers) and reservoirs for the virus (livestock) should be investigated further.
